# Hallmarks of Aging in Macrophages: Consequences to Skin Inflammaging

**DOI:** 10.3390/cells10061323

**Published:** 2021-05-26

**Authors:** Gabriela Rapozo Guimarães, Palloma Porto Almeida, Leandro de Oliveira Santos, Leane Perim Rodrigues, Juliana Lott de Carvalho, Mariana Boroni

**Affiliations:** 1 Laboratory of Bioinformatics and Computational Biology, Division of Experimental and Translational Research, Brazilian National Cancer Institute (INCA), Rio de Janeiro 20231-050, Brazil; gabrielarapozo@id.uff.br (G.R.G.); pahporto@gmail.com (P.P.A.); leandrobiomed@gmail.com (L.d.O.S.); 2 Genomic Sciences and Biotechnology Program, Catholic University of Brasilia, Brasilia 70790-160, Brazil; leane.perim@gmail.com (L.P.R.); juliana@oneskin.co (J.L.d.C.); 3 Faculty of Medicine, University of Brasilia, Brasilia 70910-900, Brazil; 4 Experimental Medicine Research Cluster (EMRC), University of Campinas (UNICAMP), Campinas 13083-970, Brazil

**Keywords:** immunosenescence, age-associated diseases, aging

## Abstract

The skin is our largest organ and the outermost protective barrier. Its aging reflects both intrinsic and extrinsic processes resulting from the constant insults it is exposed to. Aging in the skin is accompanied by specific epigenetic modifications, accumulation of senescent cells, reduced cellular proliferation/tissue renewal, altered extracellular matrix, and a proinflammatory environment favoring undesirable conditions, including disease onset. Macrophages (Mφ) are the most abundant immune cell type in the skin and comprise a group of heterogeneous and plastic cells that are key for skin homeostasis and host defense. However, they have also been implicated in orchestrating chronic inflammation during aging. Since Mφ are related to innate and adaptive immunity, it is possible that age-modified skin Mφ promote adaptive immunity exacerbation and exhaustion, favoring the emergence of proinflammatory pathologies, such as skin cancer. In this review, we will highlight recent findings pertaining to the effects of aging hallmarks over Mφ, supporting the recognition of such cell types as a driving force in skin inflammaging and age-related diseases. We will also present recent research targeting Mφ as potential therapeutic interventions in inflammatory skin disorders and cancer.

## 1. Introduction

Aging is a time-dependent progressive accumulation of significant cellular and tissue changes, including physiological, structural, and functional changes, leading to functional disorders and increased vulnerability to death [1]. This process is associated with molecular events such as genomic instability, telomere attrition, epigenetic alterations, loss of proteostasis, deregulated nutrient-sensing, mitochondrial dysfunction, cellular senescence, stem cell exhaustion, and altered intercellular communication, which can be termed “hallmarks of aging” [2,3].

The skin is our largest organ and constitutes a protective barrier that prevents excessive water loss and the entry of harmful substances and pathogens from the environment. Its aging reflects both intrinsic (or chronological) and extrinsic (such as radiation and pollution exposure) aging processes at the molecular and phenotypic levels [4]. Skin aging is a process accompanied by changes that alter the local microenvironment, such as weakening of the skin barrier and the accumulation of stressed and senescent cells, both of which foster inflammation through the invasion/release of Pathogen- and Damage-Associated Molecular Patterns [5]. The consequences of such an altered microenvironment include the promotion of the senescence-associated secretory phenotype (SASP), compromising tissue renewal and function, altered cellular interactions [6], and chronic low-grade inflammation [7]. This sterile inflammatory state, termed inflammaging, develops in several organs with advanced age and is associated with persistent inflammation that ultimately exhausts the skin’s defense system [5,8].

Macrophages (Mφ), a group of heterogeneous and plastic cells, play a central role in tissue homeostasis and repair, as well as host defense [9]. In the skin, Mφ can be found in different layers, being classified as recruited Mφ originating from monocytes following a recruitment process started by tissue injury, or as tissue-resident macrophages (TRM), which are derived from both adult and embryonic progenitors [10]. In the interfollicular epidermis, there are the Langerhans cells (LC), which can migrate to the lymph nodes to present antigens, being related to antimicrobial immunity, immune surveillance, and contact hypersensitivity [11]. Due to the shared characteristics with dendritic cells (DCs), LC have long been classified as such [12,13]. Nevertheless, after further ontogeny studies have demonstrated that LC arise from embryonic precursors and are maintained within the epidermis by local self-renewal under steady-state conditions, LC are currently considered a specialized subset of TRM [14]. Mφ located in the dermis, on the other hand, are called dermal Mφ and are associated with tissue repair and clearance [15].

To exert such a variety of functions, Mφ may acquire different phenotypes in response to various stimuli. In this sense, based on in vitro assays, Mφ have been divided into two groups based on their polarization phenotypes: M1 and M2. Classically activated Mφ are deemed as M1 and constitute catabolic, proinflammatory cells that are involved in antimicrobial host defense. M2, or alternatively activated Mφ, are anabolic cells with anti-inflammatory and tissue repair properties [16]. However, mainly due to recent advances in single-cell RNA sequencing (scRNA-Seq), it is now clear that such a dichotomy does not accurately represent Mφ in vivo but represents the extremes of a wide range of continuous phenotypes which have been reported [17,18].

The aging process has a great impact on Mφ, including alterations in Mφ metabolic and immune function, impacting the Mφ capability of clearance and immunosurveillance, constituting an important aspect of immunosenescence [19]. In fact, old Mφ in a mice model were characterized with a senescent, proinflammatory profile [20], associated with increased oxidative stress, compromised antioxidant defenses, and impaired function [21].

Interestingly, the number of LC in the skin and their capacity to migrate to the lymph node and stimulate T cells seems to be reduced in aged subjects compared to young ones [22]. In aged mice, the same process is observed, accompanied by a decline in LC maturation, but not in LC proliferation and survival levels, suggesting either a deficiency in bone marrow-derived LC progenitors or the generation of progenitors that are less responsive to chemokine and cytokine signals [22]. The same study has also described a higher level of phagocytosis in Mφ from older mice [22], which is probably a result of an age-related Mφ hyperfunction, since during the aging process the skin barrier weakens, favoring the pathogen’s invasion and stressed and senescent cells that should normally be eliminated are accumulated [23].

Mφ are considered as gatekeepers of tissue homeostasis and integrity, constituting primary inflammatory cytokine producers, as well as initiators and regulators of inflammation, and representing one of the main cellular players in adaptive immunity exacerbation and exhaustion during aging [24,25]. With that being said, it is possible to consider Mφ as important players in the promotion of chronic proinflammatory-associated pathologies, such as psoriasis [26,27], rosacea [28,29], vitiligo [30,31], and skin cancer [32,33]. In recognition of the age-related alterations on Mφ function and their importance during skin aging, in this review, we will dissect how aging hallmarks may alter the Mφ phenotype and function and connect these plastic cells with skin inflammaging. We will also present recent research targeting Mφ as potential targets for therapeutic interventions in chronic age-related skin disorders.

## 2. Hallmarks of Aging and Macrophages

**Figure 1:**
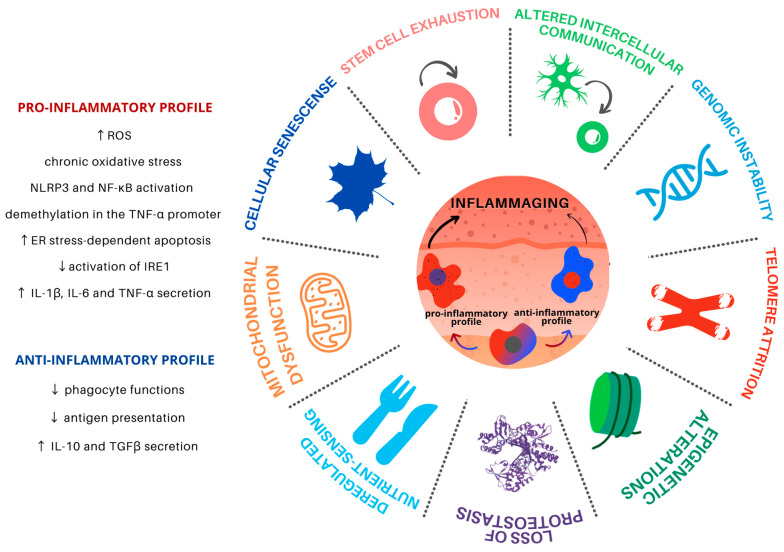
Hallmarks of aging in Mφ in the skin microenvironment. Skin inflammaging is fostered by different yet interconnected and synergistic aging hallmarks. Mφ are plastic cells that play a pivotal role in the immune system and have been associated with the persistent chronic inflammation levels found in aged skin. Skin inflammaging is characterized by a shift towards pro-inflammatory Mφ phenotypes, which promote further tissue inflammation in the skin microenvironment through the secretion of pro-inflammatory cytokines, activation of important inflammatory pathways, and increased oxidative stress. Chronic low-grade oxidative-inflammatory stress during the aging process is a key factor that stimulates a vicious cycle, contributing to age-associated disease onset. At the same time, the reduction of Mφ with an anti-inflammatory phenotype contributes to the decrease in antigen presentation and phagocytosis, contributing to tissue homeostasis disturbance.

Aging is a progressive and common process for all cells and tissues and can be caused by both intracellular and extracellular factors. It leads to organismal dysfunction on multiple levels, the main underlying processes being identified as the hallmarks of aging (Figure 1). Such hallmarks are interconnected and converge to tissue inflammation and dysfunction [5].

### 2.1. Genomic Instability

DNA damage accumulation is expected to occur with aging and accumulates as a result of many endogenous and exogenous factors. Genomic instability in the aging process can be associated with somatic mutations, copy-number alterations, and chromosome abnormalities for nuclear as well as mitochondrial DNA (mtDNA) [2]. Such DNA alterations may affect essential genes and transcriptional pathways, resulting in dysfunctional cells.

In the literature, the causes of genomic instability in Mφ are scarce and one work points out that it can be induced by pathogens such as *Mycobacterium tuberculosis* [34]. *M. tuberculosis* is the causative agent of tuberculosis with a pathological outcome associated with the formation of granulomas. The granulomas formed in the development of several chronic diseases (due to persistent inflammatory stimuli) can modulate molecular programs, which are involved in TRM differentiation and relate to clinical outcomes [35] and DNA damage [36].

Since Mφ are the most abundant immune cell type in the skin, and this tissue is directly exposed to several environmental factors, such as UV radiation, genomic instability could be a central hallmark of aging in aged Mφ; after all, numerous DNA injuries can lead to accumulated damage and altered Mφ function in this tissue [37]. This in turn can lead to altered gene expression of molecules such as cytokines, MHC class II, transcription factors, an exacerbated production of reactive oxygen species (ROS) [15], and NF-κB signaling in response to DNA damage [38], rendering Mφ more susceptible to apoptosis, impairing their phagocytosis function [39], and contributing to inflammaging [40].

### 2.2. Telomere Attrition

Telomeres protect the ends of chromosomes from degradation and abnormal recombination. Considered a primary hallmark of aging, telomere attrition causes the loss of chromosome protective structures as they gradually get shorter [2]. This shortening process has also been closely connected with inflammation [41,42].

Comparing young and old mice, Kang and colleagues (2018) observed that the shortening of telomeres in Mφ leads to increased ROS production, the same phenotype observed for genomic instability (see Section 2.1). Furthermore, in their experiment, knockout mice for Telomerase RNA Component (*Terc^−/−^*), a gene that encodes for the RNA that serves as a template for the telomere repeats, showed telomere dysfunction in Mφ, which is associated with hyper inflammation and mitochondrial abnormality, followed by oxidative stress with hyperactivation of the Nod-like receptor protein 3 (NLRP3) inflammasome (see Section 2.6) [43].

The increase in oxidative stress can induce DNA breakdown, which can lead to mutations that may explain most of the changes described in the aged Mφ. The main inflammatory signaling pathway, NF-κB, regulates the maintenance of telomeres and telomerase activity [44], just as the latter regulate NF-κB activity [45]. This relationship leads to a defective autophagic response and overexpression of inflammatory cytokines, such as TNF-α, IL-6, and IFN in circulating Mφ [46].

### 2.3. Epigenetic Alterations

Epigenetic alterations involve changes in DNA methylation (DNAm) patterns, post-transcriptional modification of histones, and chromatin remodeling [2,47]. These aging-induced epigenetic changes in Mφ are mainly responsible for controlling the inflammatory profile and cell differentiation [48,49].

DNAm undergoes predictable time-dependent modifications across CpG islands and is influenced by both intrinsic and extrinsic processes. Molecular clocks have been developed in order to calculate the “biological age” of biological samples using methylome data [50,51], including a skin-specific Molecular Clock [51]. Age-associated epigenetic remodeling involves highly localized gain/loss of DNAm at the binding sites of transcription factors associated with the monocyte-macrophage differentiation process [52]. Despite a major lack of comprehension regarding the cause or effect role of epigenetic changes and aging phenotypes, recent studies have shed light on at least a few events that connect epigenetic changes in Mφ and age-related phenotypes, such as inflammation and differentiation.

For instance, aging-associated changes in DNAm, particularly the demethylation in the tumor necrosis factor (TNF-α) promoter, a cytokine predominantly produced by Mφ, revealed a possible link between inflammation, Mφ, and chronic age-related diseases. The promoter demethylation has been described to occur in peripheral blood leukocytes and Mφ of aging subjects and is accompanied by a reduction of TNF-α reporter gene activity [53], possibly associated with chronic inflammatory processes.

Other studies have also revealed that protein-3 containing the Jumonji domain (Jmjd-3), a H3K27 demethylase, promotes the induction of Irf-4, and SMYD-3, an H3K4 methyltransferase, of IL-4 and IL-12 [54,55]. These histone-modifying proteins play a central role in aging and their activities increase in the course of this process. In Mφ, they contribute to the positive regulation of regenerative and anti-inflammatory profiles [55,56].

These epigenetic alterations in the skin microenvironment contribute to inflammaging and can be directly linked to clinical outcomes. For instance, the chronic exposure of Mφ to inflammatory triggers and products of dead or senescent cells (see Section 2.7) can impose epigenetic changes that cause Mφ’ altered response capacity during skin aging [52].

### 2.4. Loss of Proteostasis

Proteostasis is defined as the process of protein homeostasis maintenance and comprises a complex proteostasis network (PN), mainly composed by specialized proteins such as chaperones and cochaperones, translational machinery, the ubiquitin-proteasome system (UPS), and the autophagy machinery [57]. The PN has the role of controlling protein synthesis, modification, secretion, and degradation. It also reduces misfolded proteins by restoring, removing, or degrading them through the unfolded protein response (UPR) activity to prevent their accumulation in cellular compartments [58]. The chaperone-mediated autophagy (CMA) is another player in the proteostasis balance. HSPA8 is a central component of CMA and is an abundant protein in Mφ and other immune cells. Together with a co-chaperone complex, HSPA8 recognizes “CMA-targeting recognition motifs” in the targeted protein sequence, unfolding the substrate and delivering it to a protein called lysosome-associated membrane protein 2A (LAMP2A), which internalizes the targeted proteins for subsequent degradation in the lysosomal lumen [59]. If those mechanisms fail to restore homeostasis, apoptotic pathways may be activated to ensure survival of the organism [58].

Autophagy is a paramount process in the maintenance of skin homeostasis throughout aging, the consequences of age-related autophagy decay affecting different skin types, including LC. An example describing the consequences of the loss of proteostasis on aged skin is the change of elastin, collagen, and melanin levels [60,61,62] found in wrinkled and hypopigmented skin [60,63]. The balance in the composition of those proteins is essential for skin function and health and, interestingly, Mφ play an important role in this context. For instance, Mφ synthesize metalloelastases (e.g., metalloelastase 12) that participate in the elimination of nonfunctional elastin aggregates generated in the skin as a consequence of photoaging [64,65].

If on one side, healthy Mφ are important contributors to the maintenance of proteostasis, aged Mφ exhibit diminished inositol-requiring enzyme 1α (IRE1α) activation (a stress sensor that activates UPR) and increased susceptibility to endoplasmic reticulum (ER) stress-dependent apoptosis [66]. During high levels of ER stress, UPR activates IRE1α, which in turn assists the alternative splicing of X-box binding protein 1 (XBP1) mRNA [67]. After its activation, the transcription factor XBP1 induces the expression of cytokines such as pro–IL-1β [68]. However, it has been shown that Toll-like Receptors (TLR) in mice and human Mφ can directly activate XBP1, without UPR mediation, or even in synergy with ER stress [67], leading to the splicing of XBP1 and activation of a sustained proinflammatory environment by IL-1β, IL-6, and TNF [67,69].

In fact, it has been shown that the inflammasome is activated in the context of excessive misfolded protein accumulation, which is exacerbated in autophagy- or p62 (sequestosome 1)-deficient Mφ [70,71]. In addition, ER stress can also be transferred from neighboring parenchymal cells to TRMs by upregulating the splicing of UPR components, such as *Grp78*, *Gadd34*, *Chop*, and *Xbp-1* [72,73]. This phenomenon of a “transmissible” ER stress state is mediated by the production of IL-4, IL-10, and by apoptotic bodies from stressed cells [72]. Therefore, the age-related loss of proteostasis in the skin affects Mφ and seems to contribute to the local inflammaging phenotype.

### 2.5. Deregulated Nutrient Sensing

Aging directly affects the sensors and molecular targets of nutrients and fluid homeostatic regulation [74]. One of the underlying mechanisms by which aging promotes deregulated nutrient sensing is by promoting disturbed insulin sensitivity, which compromises the capacity of some tissues to uptake and metabolize nutrients [75].

Importantly, nutrient sensing components have been directly linked to longevity, including the mammalian targets of rapamycin (mTOR) and AMP-dependent protein kinase (AMPK), the two major components of nutrient sensing and metabolic regulation. AMPK has an inhibitory effect on mTOR signaling, which is activated during nutrient starvation, leading to a rise in the AMP:ATP ratio [76]. In association with nicotinamide adenine dinucleotide (NAD), high AMP levels activate sirtuins, responsible for insulin signaling pathway and longevity regulation. Consequently, the excess of nutrient availability can promote aging-associated diseases [77,78].

Accordingly, the modulation of nutrient sensing signaling influences several immune cell types including Mφ. It has been said that the Mφ immunometabolism influences Mφ polarization and activation, processes which are tightly linked to skin homeostasis and inflammaging. Mφ metabolic signatures have been closely connected with the M1-like and M2-like phenotypes; the M1-like Mφ heavily relying on glycolysis, and the M2-like Mφ being more dependent on oxidative phosphorylation [79]. During the aging process, Mφ function and phenotypes are disturbed due to many factors, including nutrient sensing dysregulation and the installation of a chronic low-grade inflammation environment in the tissue. In this sense, deregulated nutrient sensing can increase Mφ glycolysis and suppress the oxidative phosphorylation (via attenuation of IL-4-induced anti-inflammatory responses), favoring the accumulation of M1-like Mφ in the aged skin [80,81,82,83]. The presence of M2-like profile is therefore reduced, causing skin damage and promoting the progression of age-associated diseases [84,85,86].

Furthermore, FOXO and mTOR are targets of the insulin and insulin-like growth factor 1 (IGF-1) signaling (IIS) pathway and are influenced by nutrient status, altering tissue homeostasis, and inflammation [87,88]. In a very elegant experiment, the skin of mice lacking both the insulin and IGF-1 receptor in myeloid cells was enriched in noninflammatory Mφ phenotype after the induction of dermatitis. When compared to controls, it showed evidence of a proinflammatory IR/IGF-1R-dependent pathway and a connection between cutaneous inflammatory responses and diseases such as insulin-resistant diabetes mellitus type 2 [89]. In addition, SASP has been highly associated with Mφ inflammatory factors in conditions of hyperglycaemia, contributing to the fueling of low-grade inflammation in diabetes [90]. Under nutrient starvation, FOXO1 migrates to the nucleus after phosphorylation and seems to stimulate proinflammatory TLR4 signaling and IL-8β production in Mφ. FOXO1 migration also stimulates the expression of the anti-inflammatory cytokine IL-10 in M2-like cells [91], supporting the phenotypic development of aging Mφ in distinct directions [19].

Bone marrow-derived Mφ have also shown an increasing expression of growth hormone receptor (GH-R) and GH-R-dependent induction of inflammatory components in aged mice. Current evidence suggests that the downregulation of NLRP3 inflammasome in Mφ by GH-R is capable of maintaining immune system homeostasis and extending health- and lifespan [92].

Since nutrient sensing signaling pathways can be pharmacologically modulated and are closely linked to inflammation, interesting observations could be made regarding the manipulation of Mφ phenotypes in the skin. For instance, the modulation of AMPK/mTOR/NLRP3 inflammasome signaling using Metformin revealed that the drug treatment promoted reduced NLRP3 signaling and promoted the regenerative M2-like phenotype in skin Mφ, paving a way to re-establish skin Mφ equilibrium [93]. Several other anti-inflammatory drugs target immunometabolism and may also contribute in this sense, as revised by [94].

### 2.6. Mitochondrial Dysfunction

Mitochondrial production of ROS is crucial for the skin’s defense against pathogens [95]. However, dysfunctional mitochondria in Mφ can generate excessive production of ROS and cause damage to important intracellular structures, including the mtDNA [22], contributing to defective apoptosis and activation of inflammasomes [96]. Aged Mφ present mitochondrial dysfunction associated with decreased ATP production, reduction of mitochondrial membrane potential (ΔΨm), and increased oxidative stress, as well as depreciated antioxidant defense response that can impair Mφ functions and lead to senescence [97]. For instance, during lung *Streptococcus pneumoniae* infections, impaired mitochondrial function of aged Mφ increases lung pathology and oxidative stress [98].

The activity of the oxidative phosphorylation system is also altered in aging. During aerobic respiration, oxygen can be reduced prematurely, generating a high amount of ROS, a process that is exacerbated in senescent cells [99]. Besides that, Minhas and colleagues (2019) demonstrated the importance of NAD^+^ levels to maintain mitochondrial respiration and regulate Mφ phagocytosis in an anti-inflammatory homeostatic state, both in vitro and in vivo. NAD^+^ levels can be replenished by de novo synthesis and via the kynurenine pathway. Blockage of de novo NAD^+^ synthesis impaired phagocytosis and resolution of inflammation in aged Mφ [100].

In addition to sensing and cleansing cellular debris, Mφ also detect accumulation of mitochondrial garbage in the cellular microenvironment, leading to a continuous stimulation of these cells and thus their activation [101] and thus sustaining an environment of chronic low-grade inflammation with production of cytokines and ROS [97]. ROS accumulation in intracellular microenvironments (not only in the mitochondria) can cause DNA damage in aged tissues [28]. In the context of the skin, ultraviolet (UV) radiation-induced mtDNA injury also leads to more ROS production, accelerating photoaging [102]. In photodamaged skin, xanthine oxidase-induced ROS is reported to be the cause of alterations in collagen biosynthesis in cultured human dermal fibroblasts [103]. Moreover, other enzymatic and non-enzymatic sources of ROS are observed in the skin. Besides mitochondrial ROS production via electron transport chain and UV-induced ROS, there is production of ROS via peroxisomes, ER, and skin cell membranes [104]. In cultured Mφ, Ives and colleagues (2015) demonstrated that xanthine oxidase (XO) is the major source of ROS [105]. XO expression and activity has also been shown to be increased in old mice, closely associating with oxidative stress and exacerbated ROS formation [21].

As extensively revised by Beek and colleagues (2019), there is a tight link between mitochondrial dysfunction and ER in aged Mφ, that results in impaired calcium and redox homeostasis and leads to oxidative stress and activation of several pathways, including the inflammasome. Consequently, a proinflammatory environment is promoted by enhancing IL-1β secretion and nuclear translocation of NF-kB [19]. Furthermore, it has been proposed that this age-related oxidative-inflammatory stress (“oxi-inflammaging”) occurs in a vicious cycle during aging [106]. Taken together, it can be expected that age-related ER and oxidative stresses can contribute to an enhanced production of pro-inflammatory cytokines in Mφ and thus to systemic inflammaging, favoring the onset of pathologies.

### 2.7. Cellular Senescence

Cellular senescence is a cellular state characterized by cell cycle arrest, even under growth-promoting conditions [2]. Other phenotypes of cellular senescence include apoptosis resistance and SASP [107]. In the human skin, senescent keratinocytes and fibroblasts accumulate with age and support a feedforward system mainly mediated by SASP to accelerate tissue function decay [108]. Senescent cells show increased production of proinflammatory cytokines, chemokines, growth factors and metalloproteinases [109], telomere attrition (see Section 2.2), epigenetic alterations (see Section 2.3), loss of proteostasis (see Section 2.4), and dysfunctional mitochondria (see Section 2.6), underscoring the many facets of the senescent cell phenotype.

The age-related alterations of immune system elements have been defined as immunosenescence and are characterized by changes in anatomical barriers, lymphoid organs and immune cell function, all of which synergize to result in a systemic organismal low-grade inflammation, deemed inflammaging [5].

Intrinsic and extrinsic factors of immunosenescence affect both recruited Mφ and TRMs. In homeostatic conditions, activated Mφ clear cell debris [110], but when senescent cell clearance is not effective, such cells accumulate and intensify SASP, causing several alterations in the local milieu, including Mφ dysfunction [111]. Using a mouse model, Prattichizzo and colleagues (2018) demonstrated that in hyperglycemic conditions, both cellular senescence and SASP can be induced in Mφ [90]. In aged tissues, the activated Mφ produces molecules that drive inflammatory response, such as IL-6, matrix metalloproteinases, chemokines and other mediators [112,113]. Together, these indicate that dysfunctional Mφ are both a result of dysfunctional niches and cellular senescence, in addition to contributing to the maintenance of low-grade tissue inflammation.

### 2.8. Stem Cell Exhaustion

Stem cell exhaustion is a consequence of the sum of several hallmarks of aging mentioned above and is likely one of the main culprits for the loss of tissue regenerative capacity, and consequently organismal aging. Examples of that loss have already been related to immunosenescence [114,115].

Skin homeostasis is mainly maintained by two stem cell types: dermal mesenchymal stem cells (dermal MSCs), present in the inner layer of dermis, and epidermal stem cells (ESCs), located in the basal epidermal layer. While ESCs are responsible for epidermal cell renewal, a consequence of the capacity to differentiate into different cell lineages of the skin, such as keratinocytes and melanocytes [116,117], dermal MSCs are capable of differentiating into subcutaneous adipocytes, osteoblasts, and chondrocytes [118,119]. With aging, a loss of ESC and dermal MSC production and differentiation is observed, with consequent deceleration of skin cell renewal and reduction of skin healing capacity [119,120].

Tissue-resident hematopoietic cells, progenitors of Mφ, can self-maintain independently of hematopoietic stem cells (HSC). Mφ derived from yolk-sac are replaced by HSC-derived ones only in a few organs, including epidermis skin layer (LC originating from erythro-myeloid progenitors) [121]. This statement suggests a more important role is played by the cell’s origin than its tissue location in life span [14]. As a consequence, it is not surprising that the aged skin tends to have less LC in its composition, decreasing antigen-specific immunity [122].

Wound repair is a continuous process comprising four phases: hemostasis, inflammatory, proliferative, and remodeling (or resolution) phases [123]. With the advance in aging, there is a delayed lesion reepithelization and decreased tensile strength [124,125,126]. That may also indicate the importance of the presence of Mφ subpopulations in key moments of wound skin repair [123]. The M1-like Mφ profile contributes to the early stage of skin healing by promoting an inflammatory response with the production of high levels of proinflammatory cytokines. On the other hand, M2-like Mφ are responsible for the tissue repair process itself, including the regulation of re-vascularisation processes, fibroblast proliferation, and myofibroblast conversion, in addition to collagen production via inhibition of the AMPK/mTOR/NLRP3 inflammasome signaling axis, as discussed in Section 2.5. [93]. Immune cells such as Mφ are capable of activating epidermal stem cells for re-epithelialization under the establishment of an inflammatory wound microenvironment [127].

Through single-cell transcriptomic data analysis, a study was capable of characterizing a dermal subpopulation of Mφ that contributes to local nerve regeneration and axon sprouting after a mechanical injury [128]. Another study also observed that during skin repair, Mφ are also capable of stimulating the proliferation of adipocyte precursors [129]. Aged Mφ tend to lose their ability to migrate into wounds, with consequent retention of the Mφ at the dermis and increased tissue damaging release of ROS and proinflammatory cytokines [130]. In the epidermis, such an excessive proinflammatory microenvironment depletes epidermal stem cells, further contributing to compromised skin healing capacity [131].

### 2.9. Altered Intercellular Communication

The age-related changes in intercellular communication have been characterized in the autocrine, paracrine, endocrine, and neuroendocrine levels. The neurohormonal signaling (e.g., renin-angiotensin, adrenergic, insulin-IGF1 signaling) tends to be deregulated in aging as inflammatory reactions increase, immunosurveillance against pathogens and premalignant cells declines, and the composition of the peri- and extracellular environment changes, thereby affecting the mechanical and functional properties of all tissues, including the skin [2,132].

Recently, it was observed that mast cells are important in the recruitment of Mφ in aging through the change in the pattern of chemoattractant cytokines [133]. But there have still been few studies on how these intercellular communications involve Mφ in the skin. Still, it is already known that the age-related changes in intercellular communication are associated with inflammation. The accumulation of tissue damage throughout life, the likelihood of cytokines being secreted by senescent cells, the enhanced activation of the NF-κB transcription factor, and the occurrence of a defective autophagy response all seem to foster the immune system failure [96,134].

## 3. Targeting Macrophages in Chronic Skin Diseases

**Figure 2:**
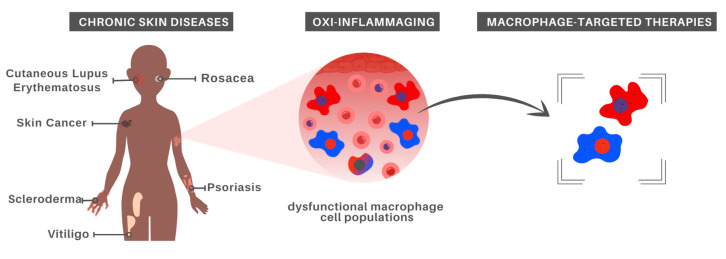
Macrophage-centered approaches for chronic skin disease treatment. In recognition of the major role played by aged Mφ in oxi-inflammaging, which in turn has been recognized as a risk factor for the development of chronic inflammatory skin diseases, it is possible that Mφ-targeted therapies constitute a promising alternative for at least some inflammatory skin disorders.

Given that aging strongly impacts immune cells, immunosenescence has been listed as a key feature in age-associated diseases arising from the imbalance between inflammatory and anti-inflammatory states. In this section, we will discuss chronic inflammation associated with different skin pathologies, highlighting the involvement of Mφ and pointing to treatment possibilities (Figure 2).

### 3.1. Psoriasis

Chronic inflammation, oxidative stress, and proinflammatory cytokines play significant roles in the onset and progression of psoriatic skin lesions, which are characterized by hyperproliferation and altered differentiation of keratinocytes, as well as high T cell and Mφ infiltration [135,136,137]. There is evidence that Mφ are highly activated in psoriatic lesions with increased production of TNF-α and classical activation of mononuclear phagocytes. Accordingly, the pathological overexpression of TNF-α is involved in the etiology of psoriasis in humans [138] and is intensified with age-related telomere attrition conditions [46]. Moreover, mononuclear phagocytes induce cleavage and release of soluble forms of CD14 and CD163, reinforcing their participation in the inflammation at the lesion site [139]. In mice, a study depleted Mφ from skin lesions and observed significant recovery in inflammation with reduced levels of TNF-α [140]. Another study showed that T regulatory lymphocytes are capable of suppressing the tissue-damaging activity of Mφ-mediated psoriasis [136]. Moreover, Piperlongumin (PPL) epigenetically inhibits histone-modifying enzymes, which include class I (HDAC1–4) and class II (HDAC6) histone deacetylases, in Mφ. In a psoriasis mouse model, PPL inhibited the hyperproliferation and inflammation of keratinocytes and Mφ, relieving symptoms and highlighting its potential use as a therapy [39]. Furthermore, given the convincing evidence that oxidative stress is involved in the pathogenesis of psoriasis, antioxidants have been suggested as potential treatment options, such as proanthocyanidins (class of flavonoids with antioxidant, anti-inflammatory, and antiangiogenic properties) [141,142]. Another example is Astilbin, a bioactive compound extracted from the medicinal herb *Rhizoma Smilacis glabrae*, which has also been shown to have the potential effect of reducing ROS and activating NRF2 (a key transcription factor in the cellular defense against oxidative or electrophilic stress), as well as inhibiting Vascular Endothelial Growth Factor (VEGF), an important factor in the maintenance of inflammation caused in psoriasis [143].

### 3.2. Melanoma

Melanoma is a rare but aggressive type of skin cancer whose etiology is related to aging, chronic exposure to UVB radiation, and genetic susceptibility [144]. Mφ have been investigated as both melanoma suppressors and supporting cells, suggesting the need for further investigation of the theme. Mφ can contribute to melanogenesis in conditions of chronic inflammation through the production of ROS and the reinforcement of oxidative stress [145], as well as through the direct interaction with melanoma cells in a way that facilitates their dissemination [146]. In addition, our group has identified a consistent, but yet not fully characterized, Mφ subpopulation that is enriched in metastatic melanomas with poor prognosis and immunosuppressed tumor microenvironment [147]. Moreover, scRNA-Seq analysis identified a gene signature of myeloid cells that facilitate metastasis in melanoma [148]. Nevertheless, due to their inflammatory activity, activated Mφ have been used in vitro and in vivo to help target melanoma cells. For instance, Mφ can be reprogrammed in vitro and injected intravenously in mice to reduce melanoma pulmonary metastases [149]. Additionally, reprogrammed tumor-associated TAM with synthetic triterpenoid CDDO-Me lead to reduced IL-6 secretion (see Section 2.2 and Section 2.3) and inhibited surface expression of CD163 [150], a Mφ marker associated with poor clinical outcomes in melanoma [151].

### 3.3. Non-Melanoma Skin Cancer

Basal cell carcinoma (BCC) and squamous cell carcinoma (SCC) are the most common non-melanoma skin cancer types, with aging constituting a strong risk factor for their incidence [152,153]. TAM play a relevant role in the pathology of both types of cancer. Although a M2-like polarization can be induced in Mφ by SCC cells in a co-culture model, [154] another study pointed to Mφ in the SCC microenvironment expressing both M1-like and M2-like markers, highlighting the heterogeneous states of Mφ in SCC [155]. Interestingly, CD68^+^ TAM are enriched in SCC when compared to BCC tumors [156]. Taken together, these results could indicate that TAM can contribute to inflammaging and can be targeted to decrease inflammatory responses.

### 3.4. Cutaneous Lupus Erythematosus

Cutaneous lupus erythematosus, or discoid lupus erythematosus (DLE), is an autoimmune disease characterized by the presence of diverse autoantibodies and chronic inflammation, mainly in the skin. The constant presence of immunosenescence and inflammation simulates an “inflammaging” milieu that renders the immune system components of patients with DLE more sensitive to the aging process, since the clinical manifestations presented by young DLE patients resemble those that occur with age-related physiological senescence [157].

As observed throughout the discussion presented in the first section of the present review, Mφ actively participates in the aging process by promoting oxidative stress. Their relationship with the pathophysiology of DLE is no different, playing an important role in DLE pathogenesis through oxidative damage-mediated lesions. Unexpectedly, such a pathological role played by Mφ does not seem strongly related to any specific Mφ phenotypes; Chong and colleagues (2015) observed Mφ displaying M1-like, M2-like, and CD163^+^ with mixed polarizations in DLE samples [158].

Despite the differences between DLE and systemic lupus erythematosus (SLE), both conditions have Mφ-mediated oxidative stress as a key factor in disease onset and progression [159]. Different studies with anti-oxidative therapies have already resulted in significant clinical responses for SLE, and we envisage that it could also apply to DLE. For instance, Lee and colleagues (2014) observed that SLE patients that responded to the rituximab treatment, which mediates the binding and phagocytosis of B cells by Mφ, had higher levels of the genes encoding MnSOD, Cu/ZnSOD, catalase, GPx-1, Gpx-4 and GR, which are necessary for cellular antioxidant capacity [160,161].

### 3.5. Rosacea

Rosacea is classified as a chronic inflammatory skin disease of the central facial skin characterized by an abnormal activation of immune responses, vascular dysfunction, and a strong alteration in skin barrier permeability [162]. The disease can be classified into four main clinical subtypes: erythematotelangiectatic, papulopustular, phymatous, and ocular rosacea. The clinical complexity in distinguishing those subtypes can be related to a possible progression from one subtype to another, in addition to their co-occurrence [163].

Early stage perivascular and later-stage pilosebaceous infiltrates, for example, are highly composed of adaptive and innate immune system cells, such as type 1 and 17 T helper cells, Mφ and mast cells in papules and erythema, neutrophils in pustules, and plasma cells in phymata [164]. Trigger factors of rosacea lead to the release of IFN-**γ**, TNF-α, matrix metalloproteinases, and IL-26, which are responsible for the migration and polarization of Mφ, in addition to Mφ IL-1 synthesis [165]. Moreover, the activation of inflammasomes in Mφ influences the expression of clinical symptoms in rosacea [166]. In this process, chronic oxidative stress is extremely relevant for maintaining the disorder, since antioxidant and oxidant status were unbalanced in patients with rosacea, when compared to controls [167]. A series of drugs that act in the short and long-term targeting of the antioxidant metabolism have been tested for rosacea, presenting a significant advance in the treatment of such patients [168].

Liu and colleagues (2020) demonstrated that there is a modulation of M1-like Mφ in a rosacea mice model via one ADAM-like metalloproteinase activation, ADAMDEC1, exclusively expressed in Mφ and mature dendritic cells, corroborating the inflammatory status of the disease [28]. Another study concluded that rosacea presents abnormal Mφ infiltration, and that the treatment with Paeoniflorin, a traditional Chinese medicine, leads to Mφ inhibition in rosacea inflammatory response [29]. Another study has tested an undirected treatment against the inflammatory microenvironment in rosacea that hampers polarization into an M1-like profile. The anti-inflammatory and anti-angiogenic properties of aspirin alleviate rosacea-like skin dermatitis via Th1 and Th17-polarized immune response suppression, inhibited NF-κB signaling, and the release of pro-inflammatory cytokines [162].

### 3.6. Scleroderma

Systemic sclerosis, or scleroderma, is a rare connective tissue disease, characterized by systemic autoimmunity and fibrosis in multiple organs, including the skin, internal organs, and even blood vessels. The pathogenesis of scleroderma has been gradually clarified and seems to involve genetic, environmental, and, more importantly, immunological factors. The activation of the humoral and cellular immune response seems to be the trigger for the inflammatory process and activation of pro-fibrotic cytokines such as TGF-β, which acts by stimulating fibroblasts to increase extracellular matrix deposition [169]. In agreement with such observations, early scleroderma skin is characterized by the presence of inflammatory cells, predominantly Mφ, but also T lymphocytes and mast cells [170]. Importantly, Mφ are a key component of scleroderma fibrosis, interacting with and activating fibrogenesis by tissue fibroblasts [171].

Despite not being considered as an age-related disorder, it is increasingly clear that systemic sclerosis is aggravated by advanced age [172]. In this sense, different aging hallmarks have been studied in scleroderma patients; immunosenescence being hypothesized as an age-related process which influences the disease outcome [172]. Nevertheless, the underlying mechanism still requires further investigation. SASP has also been suggested as a mechanism by which the age-related accumulation of senescent cells promote Mφ activation and tissue fibrosis in scleroderma patients [173].

Studies in general have highlighted the role of Mφ in the development of fibrosis in the skin and other organs, such as the heart, since age-related changes in the composition, gene expression and functionality of cardiac tissue Mφ render the tissue more susceptible to fibrosis [174]. Corroborating the important role played by Mφ in scleroderma, different in vitro and in vivo experimental approaches targeting the inhibition of phosphodiesterase 4, adenosine pathway, and profibrotic growth factor receptors all seemed to confer positive benefits through the restriction of M2-like Mφ. The modulation of profibrotic growth factor receptors by nintedanib was further confirmed in a clinical study [171]. Interestingly, the modulation of M1-like Mφ differentiation also seemed to be beneficial in a murine model of scleroderma [171].

Taken together, different literature reports suggest that the fibrotic process in scleroderma is accelerated by the recruitment of Mφ and that this process seems to be aggravated by different age-related phenotypes, including immune dysfunction and cellular senescence.

Oxidative stress is another pathological feature of systemic sclerosis, acting in the path of inflammation, autoimmunity, and fibrosis. In fact, when fibroblasts derived from fibrotic and non-fibrotic skin of systemic sclerosis (SSc) patients are evaluated, higher levels of ROS are observed when compared to fibroblasts derived from healthy samples. The stressed fibroblasts promote a profibrotic phenotype through the oxidative inactivation of PTP1B (protein tyrosine phosphatase), which leads to the increase of collagen levels in the skin [175]. However, intravenous administration of NAC (N-acetylcysteine), a free radical scavenger and precursor to the antioxidant glutathione, inhibited collagen synthesis and fibroblast proliferation [176] and reduced the production of peroxynitrite by lung macrophages in vitro [177,178].

The relevance of Mφ as therapeutic targets of scleroderma is under investigation but seems promising, including at least one clinical trial in the theme.

### 3.7. Vitiligo

Vitiligo is an autoimmune skin chronic disease in which melanocytes are destroyed [179]. The development of vitiligo occurs in parallel with the activation and infiltration of immune (mainly mononuclear) cells in the skin, but with changes and losses in its functioning and effectiveness [180]. At the molecular level, ROS have been described as responsible for damaging the manifested melanocytes, leading to the generation of autoantigens through different pathways that promote a chronic oxi-inflammation environment [181].

In this context, monocyte-derived Mφ have been shown to play a relevant role in contrast to the TRM. This has been demonstrated mainly through studies that evaluated Mφ migration inhibiting factor (MIF), a pro-inflammatory cytokine whose secretion was induced by DNA damage oxidative stress [182]. Farag and colleagues (2018) observed that the concentration of serum MIF was higher in subjects with vitiligo, constituting a possible biomarker of disease severity [31]. In another study with a larger patient cohort, similar observations were made regarding MIF levels, with the difference that MIF levels were also associated with patient treatment status [183]. The serum levels of granulocyte-macrophage colony-stimulating factor (GM-CSF), essential for the development of immune system stem cells, were also found increased in patients with vitiligo, indicating the role of these cells in vitiligo pathogenesis [30].

Mφ phagocytize deposits derived from melanocytes, essential for the vitiligo recoloring process after treatment with corticosteroids [184]. In contrast, the inflammatory process induces the migration of cytotoxic T lymphocytes and, consequently, a state of anti-inflammatory imbalance. Observing the effects of PAPLAL, a nanocolloid of platinum (Pt) and palladium (Pd) that acts in the antioxidant pathway, it was suggested that it regulates the plasticity of Mφ, inhibiting M1-like polarization and favoring the balance of M2-like populations in the skin. Thus, it decreases the cytotoxic activity at the lesion site and favors the repigmentation and recovery of healthy skin [185]. Another potential treatment option for vitiligo consists of drugs such as aspirin, which inhibit the pro-inflammatory pathways and have already been seen to increase the survival of melanocytes. Aspirin has been shown to inhibit the activation of proinflammatory Mφ through the NF-κB pathway [186].

## 4. Concluding Remarks

The effect of age on Mφ function is still contentious since there is limited data on the hallmarks of aging on epidermal and dermal Mφ populations. Among the age-related changes that occur in Mφ are a heightened state of basal oxi-inflammation and diminished or hyperactive inflammatory responses, which seem to be driven by metabolic-dependent epigenetic changes. In addition, most studies conducted so far have disregarded the development origin diversity as well as polarization states associated with Mφ, underscoring the need for additional studies in these areas to fill the current knowledge gaps. Ideally, such studies should be designed to specifically investigate the aging impact over the skin’s resident or recruited Mφ subpopulations throughout the lifespan.

Due to the relevant role played by Mφ in inflammaging and other age-related phenotypes and pathologies, research in the theme is set to expand. Hopefully, as knowledge regarding the underlying mechanisms and consequences of Mφ aging increases, more therapeutic alternatives may be hypothesized and tested to promote healthy aging and longevity.
